# Road map for capacity building and development for biomedical sciences in Kenya

**DOI:** 10.11604/pamj.2020.35.94.17563

**Published:** 2020-03-30

**Authors:** Bartholomew Nyangahu Ondigo

**Affiliations:** 1 Department of Biochemistry and Molecular Biology, Egerton University, Nakuru, Kenya; 2 Centre for Global Health Research, Kenya Medical Research Institute, Kisumu, Kenya; 3 Laboratory of Malaria Immunology and Vaccinology, National Institute of Allergy and Infectious Diseases, National Institutes of Health, Bethesda, MD, USA

**Keywords:** Biomedical sciences, training, mentoring, capacity building, career and university, Kenya

## Abstract

There is an effort to develop a critical mass of biomedical researchers in low middle-income countries by funding organizations and academic institutions in high-income countries. This involves providing short- and/or long-term training. Short-term training encompasses acquiring competencies in any or a combination of fieldwork, proposal/grant writing, laboratory techniques, data management, statistical approaches for data analyses and dissemination of research findings. Long-term training involves acquisition of an array of competencies that results into an award of a Master’s or PhD degree or acceptance into post-doctoral training programs. The author is motivated to write this article to create awareness on this capacity building effort and more importantly provide much needed guidance to potential graduate students considering pursuing long-term training careers in biomedical sciences and global health from Kenya.

## Essay

### Introduction

Biomedical science combines the field of biology and medicine in order to advance the health of both humans and animals. It is designed to apply biological sciences to advance our understanding of disease and provide research opportunities into some of our most troubling health issues. Biomedical research is translational crossing barriers between basic and clinical research and applying findings from basic biomedical sciences to prevent, predict or cure disease [1]. Graduates of the program are poised to make valuable contributions in biomedical research in graduate school and beyond. In Kenyan universities, post-graduate programs encompassing biomedical sciences require at least two years for a master's or three years for a Doctor of Philosophy (PhD) for full time studies before a graduate student graduates [2]. A master's degree in a discipline in biomedical science is offered by coursework (lectures and tutorials) and examinations in year one of study, and proposal development, supervised research and thesis-write up in year two of study. Unfortunately, in a number of Kenyan universities, graduate students in their first year of study find this phase of academics to be straight-forward as it tends to place emphasis on sitting and passing exams as it lacks exposure to practical work. However, without considering how these classes can be integrated into their second year, which encompasses a wider focus on research including defining a research project, few graduate students complete their studies in time. Similarly, acquisition of a PhD degree in a field in biomedical sciences involves course work and examination, proposal development, supervised research and thesis write up that provides a significant contribution to the field within a study period of 3- 5 years. Indeed, graduate completion rate in both programs is worrisome. I write this article to provide insights to potential graduate students pursuing careers in biomedical sciences towards becoming successful researchers. A three-stage approach has been used to write this article: (a) searching the literature for capacity development in biomedical sciences and global health research in low middle income countries (LMICs); (b) analyzing and synthesizing best-practices to guide the framework of capacity building in biomedical sciences; and (c) adapting the framework into operational guidelines in a tabular form that meets the specific needs of capacity development in biomedical sciences in Kenya.

### Methods

Multiple literature sources were utilized including web site of World Health Organization (WHO) [3], PubMed, manual identification of unpublished literature sources, university theses, and conference papers that were conducted at Kenya Medical Research Institute, Kisumu and Egerton University, Nakuru. These institutions have libraries with archives of information.

### Which Kenyan university should you enroll for your masters or PhD studies in Biomedical Sciences?

Long-term trainees need to know what factors to consider when choosing universities to enroll for their long- term training. The specific factors are a function of the individual as well as the broad academic environment. Individual factors include the field of interest (specialization) within biomedical sciences that the candidate has passion in for advancement of studies. The broader environmental factors to consider include choosing either an established (high profile) versus a newly accredited university, performing a background search of the amount of funds allocated to the research portfolio to graduate students and staff in the university, availability of local prominent research professors and or research scientists to act as in-country mentors, physical location of the university in the light of terrorist threats, civil unrest, and university size. The local prominent research scientists would act as role models for the upcoming scholars and as such would encourage them to take up careers in biomedical research. Established universities are more than 20 years old, are large, complex and tend to have a strong research profile in addition to having a history of providing university education. Newly accredited universities are relatively small, simple and some could be still struggling to establish their research portfolios. As regards biomedical science research, a university with a strong linkage with local and international research institution(s) and that has dynamic faculty members whose turn-around time of reviewing and marking theses or manuscript is relatively short is the best choice. Hence, thoughtful decisions are required to guide your decisions in making the right choice largely influenced by where you anticipate being in 5, 10 and 20 years after graduate school (career development plan). Above all, each university is different and unique in its own way and its contribution to the economy and society is paramount.

### How do graduate students with their respective in-country mentors develop research partnerships with principal investigators (PI) from high-income countries (HICs)?

Mentors or principal investigators (PIs) from high-income countries (HICs) have defined research interests. Their areas of research interests can be known by searching their published work in PubMed, Scopus, Google scholar, their university's website or other search engines. The search will enable you to know the journal(s) where he/she publishes research work and his or her current and potentially future research interests. To work with the mentor, you need to identify research gaps or be part of his or her team to learn new techniques and ideas that you can later apply to bridge gaps in his or her research work. As a Kenyan graduate student, you must be ready to understand the track record and research approaches of the prospective international mentor and be ready to align your expectations with the mentor [4, 5]. For instance, for a laboratory based graduate student these could include: knowing the types of assays performed in the laboratory? What is the level of laboratory infrastructure? What are his/her strengths? How are his/her former students progressing in academic and research work? You may not find answers to all these questions; hence, you should communicate with the mentor's former and current mentees. International mentors need to also understand the Kenyan system of universities, colleges and research institutes where the graduate student (mentee) will be matriculated. An international mentor needs to be knowledgeable about the university needs based on where his/her students will enroll and the different expectations of the university in which the student is registered. Another way, a mentee can know more about a mentor is requesting for an internship position or volunteering in the mentor laboratory. As an intern, one can gain hands on practice with laboratory techniques, clinical and field research. Also, it is important to discuss your research thoughts with the laboratory and field research team as they are likely to share their insightful suggestions with or even allow you to perform basic tests to generate preliminary results. This may form part of your future discussions with the mentors. During the face-to-face discussion or email communications with the potential in-country mentor, the mentee needs to be clear and precise on his/her research thoughts. As a graduate student, you need to have a brief concept note with hypotheses and objectives of your proposed study. Also aim to convey your current and future research interests during the discussion. To increase your chances of getting a good mentor, approach several mentors. Ultimately select a mentor, you believe will suit your requirements based on your career development plan when several mentors were approached. This has the benefit of having a mentoring relationship that will get off to a good start. As a mentee you will also need to think of identifying with a well-managed, trained and active research group characterized by active peer review publications. This will ultimately contribute to your active participation and being enthusiastic as a team member. Talking to your local university professors and lecturers will be helpful as regards to linking you up with international global health mentors. This will assist you to come up with an interesting and original study proposal that is likely to be conducted in the mentor's laboratory.

### How do you ensure that you optimally utilize your short- or long-term training or fellowship?

At the beginning of your training period, you ought to establish an effective and reliable means of communication with your international and in-country mentors. Regular Skype, WhatsApp social networking messenger and e-mail communications are highly recommended for the international mentor and face-to-face discussions with in-country mentor. These discussions should be geared towards developing consensus on the research questions and the type of design to be used to test your study hypothesis. The trainee is expected to develop a clear road map of targets and expectations. For example using a PhD program, expectations and achievements could be broken down by academic semester (Table 1). By comparing your actual academic/research experiences at university or research center with the components itemized in the guidelines, you will determine if sufficient progress is being achieved. If no substantial progress is being achieved, deficiencies in the system need to be clarified. You will also be expected to develop a logical plan of personal responsibility, and schedule of activities side by side with the expected dates of completion (i.e. milestones and timeline). For instance a 12-month fellowship would have to begin with seeking institutional review board (IRB) approval, developing standard operating procedures (SOPs), initiating and completing data collection, and performing analyses and manuscript writing before completion of the fellowship. Adhering to the schedule will result in the enhancement of successful completion rates for participants in training programs. Both international and in-country mentors must assist graduate students to develop a feasible training program so that failure to meet targets can be corrected in time. Graduate students also need to be encouraged to submit progress reports that detail a logical work plan on what has already been achieved and the tasks to be done in the next phase of their studies.

**Table 1:** Guidelines for a study plan for a 3-year PhD program in a field in biomedical sciences in Kenya

Time frame	Activity
Semester 1	Register in the department and academic division/SGS (School of Graduate Studies) (Local University)
	Attendance/review of relevant courses, including cross-cutting courses – research methodology; proposal writing, scholarly writing, ethical approval, skills training (Local University/Research Institution)
	Work on proposal development
	Refine the draft proposal/Working title of research project
	Attend courses (if required at University) and seminars/journal club (Research Institution)
	Develop study plan and research instruments with guidance from supervisor(s)
	Review of the Draft proposal by supervisor(s)
Semester 2	Registration for the semester at the Department and graduate school/Presentation of progress report (Local University)
	Attendance of discipline specific journal club (Research Institution)
	Development of full proposal and presentation to the department and submission to SGS (Local University)
	Submission of full proposal to the School or Faculty (Local University)
	Revision/Review of the study plan
Semester 3	Laboratory / fieldwork, data collection
	Presentation at seminars – presentations of relevant papers
	Review of study plan/progress report
	Attending training workshops/short courses
Semester 4	Registration for the semester at the Department and graduate school/Presentation of progress report (Local University)
	Continued field/ lab work
	Data analysis
	Presentation in a seminar/conference – Draft manuscript
	Review of study plan
	Teaching experience/Teaching Assistant (Local University)
	Presentation of the first paper
	Publication of the first manuscript
Semester 5	Registration for the semester at the Department and graduate school/Presentation of progress report (Local University)
	Development of a draft or outline of thesis
	Commencement of the thesis writing process/ Data analysis
	Presentation at seminar/Attending a conference.
	Review of study plan/progress report
	Teaching experience (Local University)
	Presentation of a second paper and publication of a second manuscript
Semester 6	Finalizing of thesis writing
	First thesis draft
	Second thesis draft
	Submission of final thesis (Local University)
	The Examination process
	Public (open) defense

### What are the major hindrances of graduate students towards timely completion of their research projects in Kenya?

There are several impediments that inhibit graduate students in Kenya against timely completion of their studies. Some of the reasons cited from a graduate student perspective include: limited research funds which causes some students to secure bank loans to fund their studies and research projects or through back-breaking savings, loss of academic interest leading to deferment of studies, inadequate academic study-leaves for working students, mentor's rigidity and rivalries. From the mentor's perspective the key challenge is the shortage of trained faculty with career structure and infrastructural support [6]. The few mentors available are overwhelmed with the number of students under their mentorship, excessive number of course units they teach, examine, mark and grade require extra time that could have been used to undertake research and write manuscripts. Additional challenges for graduate students include slow internet, limited number of computers, lack of statistical analysis software at graduate student libraries, inadequate teaching of some units, for instance biostatistics and methodological research skills, and the unavailability of office stationery and photocopiers and a reduced number of access to peer-reviewed journals in Kenya for their references coupled with difficulties in obtaining recent and relevant books and journal articles (Table 2). These barriers and challenges ultimately interfere with the student's ability to complete their doctoral or master's projects. These challenges ultimately result in some students prioritizing their thesis write up and submission while their research mentors prefer manuscripts and publications for renewal of their grants and promotion. During thesis write up, the academic mentor will play the leading role as opposed to the research mentor. Contrary, the research mentor will play the leading role in manuscript writing and journal selection, although in some settings the same mentor may serve both roles. Statistical analysis within research projects can be resolved by developing multi-disciplinary synergy between staff affiliated to biostatistics division in universities in HICs and biomedical researchers working in institutions in Kenya and vice versa.

**Table 2:** Challenges encountered by graduate students and trainee fellows in Kenyan universities and research institutions

Challenge	Effect	Possible Solution/Action
Lack of motivation to study global health at graduate level	Small number of students opting to pursue global health graduate education compared to Master’s in Business Administration (MBA)	Investment by governments and their partners to improve local production of staff at these levels
Loss of interest in between studies (drop out of trainees)	Incomplete research projects	Setting up a selection interview panel when recruiting trainees to ask cross cutting questions in order to gauge the students motivation and passion for research
Inadequate language skills	Takes time to translate the idea into a written form	Encouraging trainees to take up language proficiency courses
Supervisor/mentor rivalry and rigidity	Contradicting verdicts during thesis examination	Mentorship clinics for mentors and developing standardized thesis examination criteria. Use of in-country’s mentor’s international networks to examine thesis.
Inadequate number of and experienced in country mentors	Overwhelmed mentors by graduate students	Increase of mentor and long term trainee training. Improve the mentors’ career development programs.
International mentor	Slow turn around time of responding to review of manuscript to be submitted	Trainee to inform the mentor in advance that he/she will send some work so that the mentor can block off time for that work. Mentor to give trainee a deadline when she/he will have reviewed.
University	Inadequate physical infrastructure- deficiencies in computing resources, Inadequate integration of theory and practice	Investment by governments on institutions of higher learning.

### What next after acquisition of the terminal PhD degree or completion of a global health fellowship in Kenya?

Relatively few options exist for researchers in Kenya after completion of their terminal degrees. The first option is to take up a faculty position in a department with biomedical sciences program or a related field at a local university and continue with their research collaboration with your mentor. Faculty position involves teaching and mentoring both undergraduate and postgraduate students, examining postgraduate theses, etc. This requires one to strictly budget and balance time for teaching and doing research. Firmness in time management is required and is an important skill to learn during the process of one's doctoral studies. The newly employed PhD candidate bring freshness to respective university departments and could be an additional vital human resource that would help in revising the curricula for biomedical science courses to reflect recent advances in the field of biomedicine. Secondly, taking up a full time post-doctoral position - so that you apply learnt techniques at the doctoral training to bridge research gaps in different mentor's laboratory. This could also enable you to advance your research skills, learn new assays and interact with new set of mentors who will ultimately contribute to your research capacity development. Most post-doctoral positions are available in universities and research institutions in HICs; however, it is encouraging that African institutions, for instance the African Academy of Sciences (AAS) and New Partnership for Africa's Development (NEPAD) Agency have set goals of revolutionizing science in Africa. NEPAD and AAS have developed the Alliance for Accelerating Excellence in Science in Africa (AESA), an agenda setting and funding platform established to address Africa's health and development challenges. AESA has developed innovative postdoctoral fellowships and schemes for experienced scientists which include: Climate Impact Research Capacity and Leadership Enhancement (CIRCLE), Developing Excellence in Leadership, Training, and Science (DELTAS Africa), Regional Initiative in Science and Education (RISE), The African Postdoctoral Training Initiative (APTI) and Future Leaders - African Independent Researchers (FLAIR). These represent several important programs supporting post-doctoral candidates in Africa (African Academy of Sciences) [7]. Thirdly, some graduates may opt to enroll for professional courses such as project planning and management, research bioethics, leadership skills in a specialized field in accredited universities and colleges. Indeed, this option is important because in most cases after long-term training, a recently graduated PhD trainee is often tasked to head a department or to be a full/part-time faculty at local universities or work in private sector, international agencies and the non-governmental organizations (NGO) community. The downside for this option is that highly trained biomedical scientists ultimately find themselves doing routine administrative jobs, which have little or no bearing on their research training due to lack of proper career structures within medical schools or biomedical research institutions. Lastly, applying for a joint appointment as a visiting scholar in HIC universities and research institutions while also affiliated to Kenyan institution would help to strategically promote biomedical and global health research agenda. Ultimately, this would promote active institutional collaboration between researchers from HICs and Kenya.

## Conclusion

Potential graduate students and trainees require mentorship toward a successful career in biomedical sciences. Challenges faced by graduate students in Kenya require innovative solutions to address them. Lastly, sustained engagement of successful Kenyan mentees is required to enable them acquire requisite capacities, transferable skills, and networks to propel them to become successful biomedical science global health researchers.
